# Natriuretic peptide signaling as a therapeutic target in POTS: physiological opportunities and caveats

**DOI:** 10.1007/s10286-026-01194-4

**Published:** 2026-02-17

**Authors:** Jens Jordan, Dominik Pesta, Cedric Moro

**Affiliations:** 1 Institute of Aerospace Medicine, German Aerospace Center (DLR), Linder Hoehe, 51147 Cologne, Germany; 2 Medical Faculty, University of Cologne, Cologne, Germany; 3 Cologne Excellence Cluster on Cellular Stress Responses in Aging-Associated Diseases (CECAD), University of Cologne, Cologne, Germany; 4 Institute of Metabolic and Cardiovascular Diseases (I2MC), INSERM, Toulouse University, UMR1297, Toulouse, France

**Keywords:** Natriuretic peptides, Postural tachycardia syndrome, POTS, Insulin resistance, Oxidative metabolism, Exercise intolerance, NPR1

Postural orthostatic tachycardia syndrome (POTS) is a heterogeneous disorder characterized by orthostatic intolerance, excessive upright tachycardia, reduced exercise capacity, and impaired quality of life. Many patients, particularly those with severe or persistent symptoms, do not achieve adequate improvement with lifestyle measures or existing pharmacotherapies. Consequently, there is growing interest in mechanism-based therapies targeting physiological contributors to orthostatic intolerance. A recent development is the initiation of a first-in-human clinical trial of the natriuretic peptide receptor-1 (NPR1) antagonist REGN7544 (ClinicalTrials.gov Identifier: NCT06593600), designed to restore central blood volume by reducing natriuretic peptide signaling. The emergence of such approaches makes it timely to consider the broader physiological implications of modulating this pathway in POTS.

Multiple studies have documented abnormalities in blood volume regulation in a subset of patients with POTS. Investigations using dye dilution, radiolabeled albumin, carbon monoxide rebreathing, and hemodynamic assessment consistently show reduced plasma and blood volumes [1, 2]. Other studies demonstrate small cardiac chamber size and low stroke volume, indicating reduced cardiac filling pressures and impaired venous return [3]. These hemodynamic abnormalities likely contribute to orthostatic tachycardia. They also explain why strategies to expand intravascular volume, such as high sodium intake [4], mineralocorticoids, or intravenous saline [5], can improve symptoms, although responses remain variable and often incomplete. Beneficial effects of physical exercise in POTS may be mediated in part through intravascular volume expansion [6].

NPR1, also known as guanylyl cyclase-A (GCA), the principal receptor for atrial and B-type natriuretic peptides, governs renal sodium handling, vascular tone, and extracellular fluid balance [7]. Antagonizing NPR1 represents a physiologically coherent strategy to enhance sodium retention and increase central blood volume. Clinical trials of REGN7544 POTS reflect this translational trajectory.

**Fig. 1:**
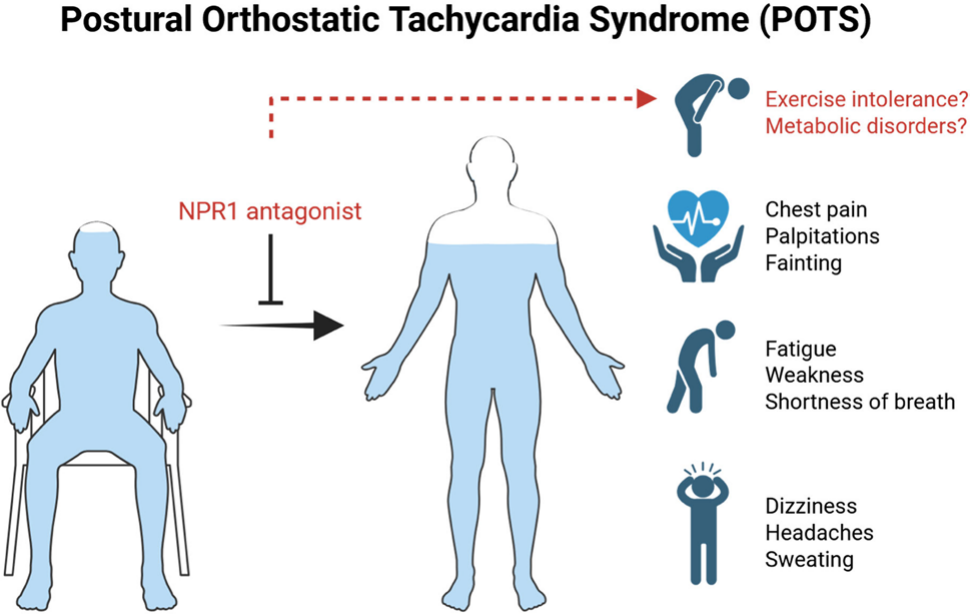
Long-term treatment of postural orthostatic tachycardia syndrome (POTS) by the natriuretic peptide receptor (NPR1) antagonist REGN7544 may lead to adverse systemic metabolic consequences and exercise intolerance. Image created with BioRender

However, natriuretic peptide signaling via NPR1 extends beyond extracellular fluid regulation [7]. A robust body of literature demonstrates that natriuretic peptides regulate systemic energy metabolism [8]. Natriuretic peptides promote lipolysis, enhance skeletal-muscle oxidative metabolism, and stimulate mitochondrial biogenesis [9, 10]. Individuals with low natriuretic peptide levels have increased risk for metabolic syndrome and type 2 diabetes mellitus [11, 12], suggesting that natriuretic peptides support metabolic homeostasis. Recent mechanistic work shows that impaired atrial natriuretic peptide signaling via NPR1 induces insulin resistance, mitochondrial dysfunction, and reduced endurance capacity [13]. The finding suggests that natriuretic peptides released during physical exertion [14, 15] mediate a training effect on muscular metabolic adaptation and improved aerobic fitness in humans. These findings are directly relevant to POTS, as patients frequently show markedly reduced peak oxygen consumption, impaired skeletal muscle oxygen extraction, low stroke volume, and abnormal metabolic responses during exercise, limitations that are major determinants of disability (Fig. 1).

In this context, therapies that reduce natriuretic peptide signaling may inadvertently compromise skeletal muscle energetics. An intervention intended to improve central blood volume could, paradoxically, worsen exercise intolerance, the very outcome clinicians aim to improve. Because diminished aerobic capacity is a defining limitation in POTS, interventions that blunt mitochondrial function or metabolic flexibility warrant careful scrutiny.

Long-term metabolic implications must also be considered. POTS predominantly affects young individuals, who may require treatment for years. Even modest reductions in natriuretic peptide activity may accumulate adverse effects on insulin sensitivity or lipid utilization over time [11]. These risks require consideration when evaluating chronic use of systemic NPR1 inhibitors.

Disease heterogeneity further complicates individualized therapeutic decision-making. Not all patients with POTS exhibit hypovolemia or altered natriuretic peptide activity. Hyperadrenergic, neuropathic, autoimmune, and mast-cell–associated phenotypes may respond differently to manipulations of sodium and volume regulation. Without reliable biomarkers, such as indices of plasma volume status, natriuretic peptide bioactivity, or renal sodium handling, systemic NPR1 inhibition may expose patients to metabolic risk without conferring hemodynamic benefit.

Future approaches might allow tissue-selective modulation of NPR1 signaling, reducing adverse systemic metabolic consequences. While theoretically appealing, such specificity is not yet achievable with available pharmacological tools. Until such selectivity exists, the broad actions of natriuretic peptides across cardiovascular, renal, and metabolic pathways remain integral to the risk profile of NPR1-directed therapies.

Given these challenges, future clinical studies of NPR1 antagonism should incorporate metabolic and functional endpoints alongside hemodynamic assessments. Early-phase trials would benefit from measures of insulin sensitivity, substrate utilization, and mitochondrial function using indirect calorimetry, exercise testing, insulin clamps, polarography, or circulating biomarkers of oxidative metabolism. Standardized assessment of exercise capacity is essential, as changes in endurance performance are clinically meaningful and directly relevant to patient well-being and mortality.

Developing targeted therapies for POTS is a timely and necessary strategy. NPR1 antagonism represents a creative and physiologically grounded attempt to address hypovolemia in selected patients. However, natriuretic peptides coordinate cardiovascular, renal, and metabolic homeostasis. Interventions that improve central blood volume may simultaneously impair skeletal muscle energetics and increase long-term metabolic risk. Recognizing and balancing these competing effects will be essential for determining whether NPR1-directed therapies ultimately provide net clinical benefit.
