# Correction: Natriuretic peptide signaling as a therapeutic target in POTS: physiological opportunities and caveats

**DOI:** 10.1007/s10286-026-01200-9

**Published:** 2026-03-07

**Authors:** Jens Jordan, Dominik Pesta, Cedric Moro

**Affiliations:** 1 Institute of Aerospace Medicine, German Aerospace Center (DLR), Linder Hoehe, 51147 Cologne, Germany; 2 Medical Faculty, University of Cologne, Cologne, Germany; 3 Cologne Excellence Cluster on Cellular Stress Responses in Aging-Associated Diseases (CECAD), University of Cologne, Cologne, Germany; 4 Institute of Metabolic and Cardiovascular Diseases (I2MC), INSERM, Toulouse University, UMR1297, Toulouse, France

**Correction: Clinical Autonomic Research**

10.1007/s10286-026-01194-4

We noticed that we utilized the wrong code name for the experimental drug discussed in the manuscript and corrected the mistake.

The original article has been corrected.

